# Identification and structural characterization of FYVE domain-containing proteins of *Arabidopsis thaliana*

**DOI:** 10.1186/1471-2229-10-157

**Published:** 2010-08-02

**Authors:** Ewa Wywial, Shaneen M Singh

**Affiliations:** 1 Department of Biology, The Graduate Center of the City University of New York, 365 Fifth Avenue, New York, NY 10016, USA; 2 Department of Biology, Brooklyn College, City University of New York, 2900 Bedford Avenue, Brooklyn, NY 11210, USA

## Abstract

**Background:**

FYVE domains have emerged as membrane-targeting domains highly specific for phosphatidylinositol 3-phosphate (PtdIns(3)*P*). They are predominantly found in proteins involved in various trafficking pathways. Although FYVE domains may function as individual modules, dimers or in partnership with other proteins, structurally, all FYVE domains share a fold comprising two small characteristic double-stranded β-sheets, and a C-terminal α-helix, which houses eight conserved Zn^2+ ^ion-binding cysteines. To date, the structural, biochemical, and biophysical mechanisms for subcellular targeting of FYVE domains for proteins from various model organisms have been worked out but plant FYVE domains remain noticeably under-investigated.

**Results:**

We carried out an extensive examination of all *Arabidopsis *FYVE domains, including their identification, classification, molecular modeling and biophysical characterization using computational approaches. Our classification of fifteen *Arabidopsis *FYVE proteins at the outset reveals unique domain architectures for FYVE containing proteins, which are not paralleled in other organisms. Detailed sequence analysis and biophysical characterization of the structural models are used to predict membrane interaction mechanisms previously described for other FYVE domains and their subtle variations as well as novel mechanisms that seem to be specific to plants.

**Conclusions:**

Our study contributes to the understanding of the molecular basis of FYVE-based membrane targeting in plants on a genomic scale. The results show that FYVE domain containing proteins in plants have evolved to incorporate significant differences from those in other organisms implying that they play a unique role in plant signaling pathways and/or play similar/parallel roles in signaling to other organisms but use different protein players/signaling mechanisms.

## Background

The FYVE lipid-binding domains were named after the first letter of the four proteins in which they were originally discovered: Fab1, YOTB, Vac1, and EEA1 [1]. FYVE proteins have primarily been associated with functions related to endosomal trafficking e.g. Hrs is involved in sorting of down-regulated receptor molecules in early endosomes [2], Vacuolar protein sorting mutant 27 phenotype (Vps27p) in endosome maturation [3], EEA1 in endocytic membrane fusion [4] and regulation of endosome-to-TGN retrograde transport via phosphatidylinositol 3-phosphate 5-kinase (PIKfyve) [5]. However, they may play other important roles in cell signaling as exemplified by Faciogenital dysplasia 1 in cytoskeletal regulation [6], Fab1p in regulation of membrane homeostasis [7-9] and Smad Anchor for Receptor Activation (SARA) [10] as well as endofin in growth factor signaling [11-13]. Structurally, FYVE domains share a fold comprising of two small double-stranded β-sheets and a C-terminal α-helix as deduced from experimentally solved structures such as the crystal structure of the FYVE domain from yeast Vps27p [14]. The fold is stabilized by eight Zn^2+ ^coordinating cysteines residues, which bind Zn^2+ ^in pairs such that the first and third pairs bind one zinc atom, while the second and fourth pairs bind the other zinc atom [14]. The FYVE domains have been characterized as phosphoinositide-binding domains that are highly specific for the phosphatidylinositol 3 phosphate (PtdIns(3)*P*) [15-18]. This ligand recognition is Zn^2+^-dependent [19] and stems primarily from a conserved ligand-binding motif, i.e. (R/K)(R/K)HHCR surrounding the third and fourth cysteine residues [14]. Mutagenesis of either the cysteines involved in Zn^2+ ^coordination or the ligand-binding conserved residues result in decreased affinity for PtdIns(3)*P *[15,19-21].

The PtdIns(3)*P*-binding signature contains three classic conserved regions: the N-terminal WxxD, the central R(R/K)HHCR and the C-terminal R(V/I)C motifs [14]. Combined they drive the PtdIns(3)*P *specific membrane recruitment of FYVE domains. However, there are several factors in addition to PtdIns(3)*P*-binding that are thought to contribute to the membrane affinity of FYVE domains: nonspecific electrostatic interactions between the basic face of the domain and the anionic membrane surface [22-24], hydrophobic interactions between the residues located in the "turret loop" near the PtdIns(3)*P *binding pocket and the membrane bilayer [14,23-26], dimerization [19,27] and pH [28]. In additional to working out the structural and functional role of various amino acids comprising the binding motifs, it has also been shown that the binding of PtdIns(3)*P *to the ligand-binding pocket of FYVE domains neutralizes nearby basic residues to reduce the local positive potential and allow conserved hydrophobic residues to penetrate the membrane interface enhancing membrane attachment [22,24,25]. Recently, a molecular dynamics simulations study explored the interactions of the EEA1-FYVE domain and verified that it undergoes a decrease in dynamic flexibility upon binding to its PtdIns(3)*P *ligand and a phospholipid bilayer [29].

The PtdIns(3)*P*-binding FYVE domains are well conserved in various organisms and have been studied extensively in different model organisms except plants. Plants possess several FYVE domain-containing proteins and PtdIns(3)*P *has been shown to be present in various compartments [30] as well as membranes [11] of plant cells. It is possible to envision that plant cells utilize the same or highly similar lipid-binding and membrane-targeting mechanisms [30] for FYVE domains given that both the FYVE domains and type III PI3-kinase, which makes PtdIns(3)*P*, are present in plant cells [31]. However some recent reports suggest that PtdIns(3)*P *may not be the only known phosphoinositide ligand recognized by plant FYVE domains, for example, the FYVE of EEA1 has been shown to be capable of binding to PtdIns(5)*P *[32,33].

We have undertaken a comprehensive examination of all FYVE domains of the model plant *Arabidopsis thaliana *(*At*) to understand the structural basis for the mechanism of their function and to explore their similarities and differences with respect to other organisms. We describe the 15 different FYVE domain-containing proteins that are expressed in *Arabidopsis*, all of which are largely unexplored. Our detailed sequence analysis and biophysical characterization of the structural models of the FYVE domains in *Arabidopsis *suggest membrane interaction mechanisms and their subtleties. Moreover, the study also reveals unique biophysical properties of plant FYVE domains, a new binding motif specific only to the variant class of plant FYVE domains and novel domain architectures unique to plant FYVE proteins.

## Results

### Identification, characterization and chromosomal localization of FYVE domain-containing proteins encoded in the *Arabidopsis *genome

**Figure 1:**
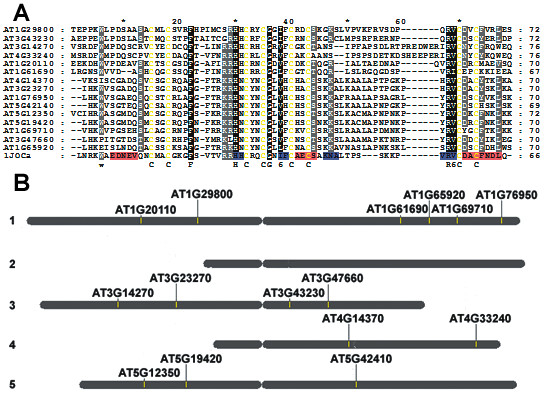
**A. Multiple sequence alignment of *At*FYVE domains**. Absolutely and moderately conserved residues are highlighted in black and grey, respectively. For comparison, the secondary structures of EEA1 FYVE domain (PDB: 1JOC chain A [37]) are highlighted in red (α-helix) and blue (β-sheet). All eight cysteine residues are colored yellow. B. A schematic localization of *At*FYVE domain-containing proteins within the *Arabidopsis *genome.

The total number of FYVE domain-containing proteins seems to be directly correlated with the total estimated number of genes for a given organism, e.g. 27 FYVE encoding genes in a total of 42,000 in *H. sapiens*, 13 in a total of 18,000 in *C. elegans *and 5 in a total of 6,000 in *S. cerevisiae *[34]. We identified 15 *At*FYVE proteins in the *Arabidopsis *protein sequence database i.e. TAIR first genome release (version TAIR 6.0, Nov 2005). Later genome releases built upon the gene structures of TAIR6 release as well as community input regarding missing and incorrectly annotated genes and they do not contain any new genes encoding FYVE proteins. Our finding of 15 FYVE proteins encoded within predicted 25,500 genes [35] of the *Arabidopsis *genome falls in line with the above observation. The initial identification was done using an automated pipeline [36]. Later, the total number of *At*FYVE proteins and their individual accession numbers were verified through manual searches performed in various databases. The 15 FYVE domains present in various *Arabidopsis *proteins (representing the entire family of *At*FYVE proteins) aligned with human EEA1 FYVE domain (PDB: 1JOC chain A [37]) are shown in Fig. 1A. Fig. 1B displays the schematic localization of the 15 *At*FYVE proteins within the *Arabidopsis *genome. The 15 identified sequences of *At*FYVE proteins are dispersed throughout the *Arabidopsis *genome, being located on all chromosomes except chromosome 2 (Fig. 1B). The disagreement of our total with previously reported totals of nine [32], over ten [38] and most recently, sixteen [39] FYVE domains stems from misannotations. For example, AT1G61620, AT1G66040, AT1G66050 and AT5G39550 proteins are all annotated as FYVE proteins but do not actually possess FYVE domains based on various sequence analysis methods.

### Domain Architecture of *Arabidopsis *FYVE proteins

**Figure 2:**
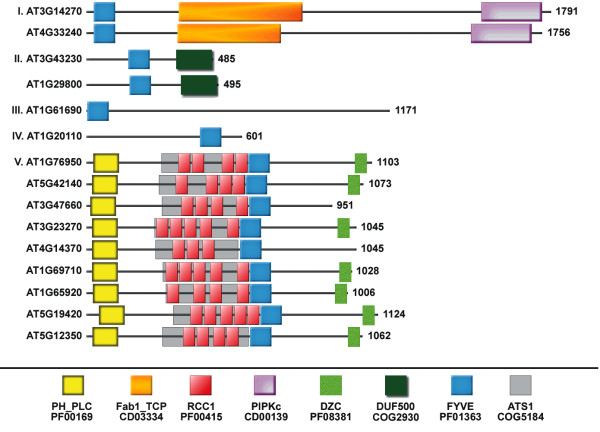
**A. Cartoon of representative examples of the five classes of *At*FYVE proteins**. The Pfam (PF), SMART (SM), Conserved Domains (CD) and Clusters of Orthologous Groups (COG) accession numbers for the different domains are given under the figure. The lengths of each protein are indicated on the right.

On the basis of domain architecture of the proteins, we propose five classes of *At*FYVE proteins (Fig. 2). Class I comprises two out of four documented *Arabidopsis *Fab1p homologues expressed in plants, i.e. AT3G14270 and AT4G33240 [40,41]. The other two Fab1 homologues do not contain a FYVE domain [40,41]. Class I members, i.e. AT3G14270 and AT4G33240, contain a FYVE domain, followed by Fab1_TCP(chaparonin-like) and PIPKc domains. AT3G14270 and AT4G33240 are annotated in NCBI database as "phosphatidylinositol-4-phosphate 5-kinase family proteins" while in UniProtKB/TrEMBL as "putative uncharacterized proteins." Our Blast analysis reveals similarity of both class I members to ppk-3 (*C. elegans*), Fab1p (*S. cerevisiae*), and phosphatidylinositol-3-phosphate 5-kinase type III (*H. sapiens*) (see supplementary material). Ppk-3 and Fab1p proteins share domain architecture identical to class I members and phosphatidylinositol-3-phosphate 5-kinase type III protein has an additional DEP domain (see supplementary material). Class II is represented by two sequences, AT3G43230 and AT1G29800, which possess two domains: a FYVE and a Domain of Unknown Function (DUF500). Class III comprises the AT1G61690 protein and class IV comprises the AT1G20110 protein. Both classes are unique in that they contain only a FYVE domain but they differ in the placement of the FYVE domain (N-terminus versus C-terminus) and also their biophysical properties (this study). UniProtKB/TrEMBL annotates function for class II-IV as putative uncharacterized. The representation of class II-IV members in the literature is full of contradictions. They are not mentioned in the classification by Drobak and Heras [38] and AT1G29800 of class II together with AT1G61690 of class III are omitted from the classification by Jensen et al [32]. Moreover, class IV protein was identified as AtAAF79901 and shown to contain a FYVE domain followed by a plant specific SGNH-plant-lipase-like domain [32]. Our analysis of the sequence suggests, however, that class IV protein is over 300 amino acids shorter than AtAAF79901, and it does not contain a SGNH-plant-lipase-like domain. Class II sequences, seem to contain an additional DUF500 domains not represented by van Leeuwen [39]. Class V is the largest class. It includes nine *At*FYVE proteins, which share similar domain architecture, i.e. Pleckstrin Homology of Phospholipase C (PH_PLC), followed by Regulator of Chromosome Condensation 1 (RCC1) regions/blades (overlapping with Alpha Tubulin Suppressor 1 (ATS1)) and FYVE domains. In addition, seven out of nine class V proteins are characterized by the presence of a DZC motif found near the C-terminus DZC. UniProtKB/TrEMBL annotates function for class V members as either disease resistance protein-like, e.g. AT5G42140 and AT4G14370, Ran GTPase binding/chromatin binding/zinc ion binding, e.g. AT1G65920, AT1G69710, AT3G23270 and AT5G12350, or putative uncharacterized, e.g. AT3G47660, AT1G76950, and AT5G19420.

**Figure 3:**
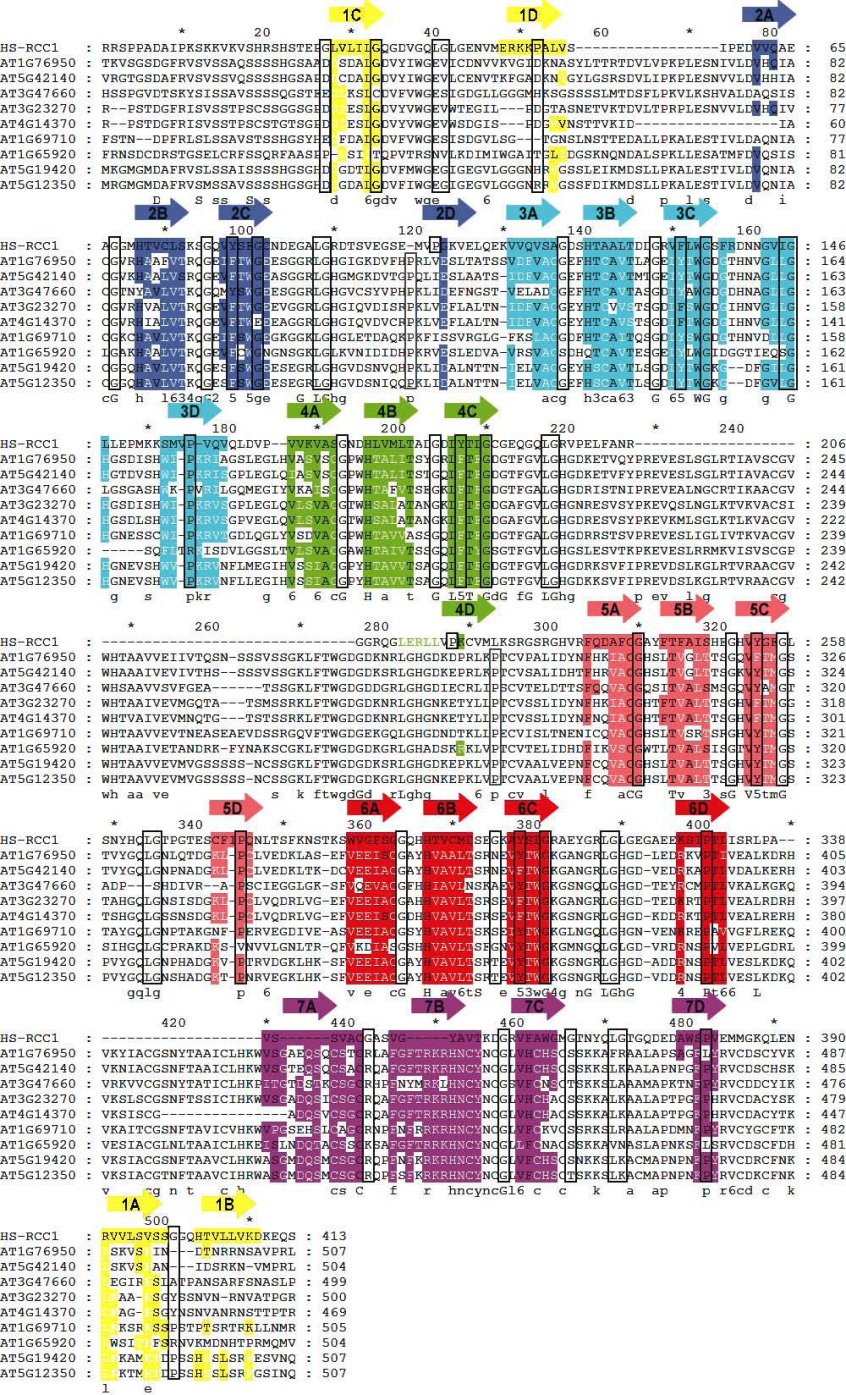
**Sequence alignment of human RCC1 and putative *Arabidopsis *homologues (class V)**. The secondary structures are adapted from solved structure of human RCC1 with minor modifications [44]. Residues absolutely conserved within the secondary structures are black on a colored background. Residues moderately conserved within the secondary structures and/or among *Arabidopsis *repeats and not human RCC1 are white on a colored background. Boxed residues correspond to amino acids which are highly conserved among each blade of the seven propeller structure [44].

The SMART database recognizes between three and five RCC1 regions within class V *At*FYVE proteins, whereas the CD-search identifies additionally yeast domain with similarity to human RCC1 domain, ATS1 domain, overlapping the RCC1 blades (Fig. 2). In some cases, only the ATS1 domain is detected by the CD-search or the number of RCC1 blades does not correspond to the number obtained from SMART database (data not shown). These inconsistencies prompted further enquiry into the number and nature of the putative RCC1 repeats identified in class V of *At*FYVE proteins. Up to now, RCC1 and RCC1-like domains that have been described are within cytoplasmic proteins associated with membrane structures, e.g. endosomes (Alsin) [42] and Golgi apparatus (HERC1) [43]. Fig. 3 shows an internal sevenfold sequence repeat of 51-68 residues present in the solved structure of human RCC1 [44] aligned with putative RCC1 regions of class V *At*FYVE proteins. In human RCC1, one half of the first sequence repeat, the C and D repeats, is made from the N-terminal end of the protein, and the other half, the A and B repeats, is made from the C-terminal end [44]. It has been suggested that this arrangement stabilize the circular arrangement of secondary structural elements through a molecular clasp mechanism similar to a belt closure [44]. Our data show that putative RCC1 blades of *At*FYVE proteins align well with six of human seven RCC1 blades. In fact, the seven highly conserved residues, i.e. four glycines, a tyrosine, a leucine and a *cis*-proline, identified in human RCC1 repeats are also mostly conserved among putative *At*RCC1 blades (boxed residues). However, it appears that the first blade of human RCC1 shares little or no primary and/or secondary sequence similarity with most putative *At*RCC1 blades. The first blade of putative *At*RCC1 may not even be a potential repeat for at least seven out of nine class V *At*FYVE proteins because they share a low sequence similarity with human RCC1 in the corresponding region as compared to other regions.

### Molecular models of the *Arabidopsis *FYVE domains and their biophysical properties

**Figure 4:**
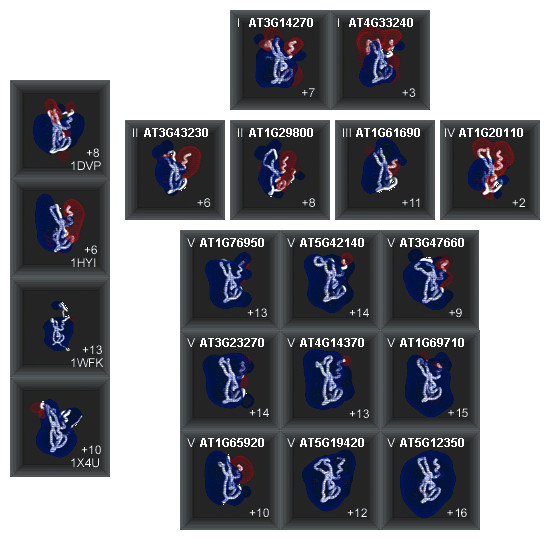
**Electrostatic profiles of FYVE domains**. Four known FYVE domain structures, i.e. PDB:1DVP, PDB:1HYI, PDB:1WFK, PDB:1X4U and fifteen *At*FYVE homology models, i.e. class I-V, are included. All FYVE domains are shown in the same orientation with the membrane binding regions facing down. The red and blue meshes represent the -1 kT/e and +1kT/e equipotential contours of the FYVE domains. The numbers in the upper left corner correspond to the *At*FYVE classification described earlier (Fig. 2). The numbers in the lower right corner correspond to total charges on each *At*FYVE domain model.

We built homology models of all FYVE domains present in 15 *At*FYVE proteins listed in Fig. 1. Since electrostatic forces play critical roles in protein-membrane interactions and numerous membrane-mediated biological phenomena, we mapped the charge distribution on the surface of each *At*FYVE domain (Fig. 4). In Fig. 4, we have constructed an electrostatic profile panel showing the location of negatively (red) and positively (blue) charged regions on the surface of *At*FYVE domains. The electrostatic profile of class I AT3G14270-FYVE model, which has a net charge of +7 (including zinc ions), bears a resemblance to the electrostatic profile of *D. melanogaster *Hrs-FYVE (PDB: 1DVP). The model of the other class I member, AT4G33240-FYVE, exhibits weaker positive potential. Both members of class II *At*FYVE domains share a potential profile that is similar to the profile observed for *H. sapiens *EEA1-FYVE (PDB: 1HYI). Class II AT3G43230-FYVE has a net charge of +6 and AT1G29880-FYVE has a net charge of +8. Intriguingly, the model of class III AT1G61690-FYVE has a very strong positive potential similar to that observed also for all models of class V *At*FYVE domains, *M. musculus *19 protein-FYVE (PDB: 1WFK) and for *H. sapiens *27 isoform β-FYVE (PDB: 1X4U). Their overall net charge is highly positive, but varies from +9 to +16. The electrostatic profile of class IV *At*FYVE model shows the weakest positive potential observed among *At*FYVE domain models. Additional electrostatic profiles for the alternative models, their PDB coordinate files and verification profiles are available online (see supplementary material).

### Sequence motifs of the *Arabidopsis *FYVE domains

**Figure 5:**
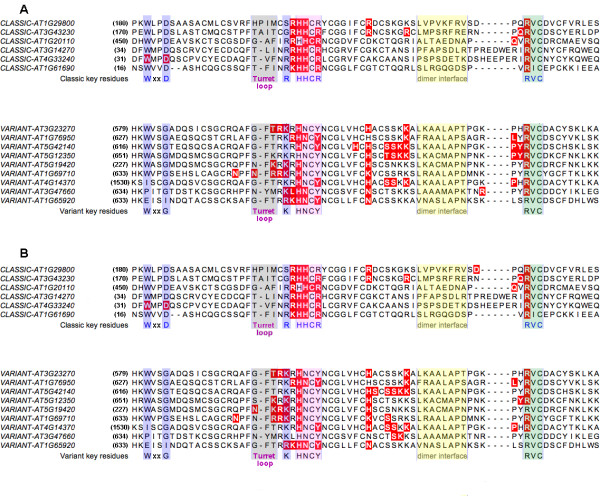
**The alignment of classic and variant *At*FYVE domains**. The conserved and variable sequence motifs of classic and variant *At*FYVE domains are boxed and highlighted in different colors, i.e. the N-terminal W and D residues in blue, the turret loop in grey, the central HHCR/HNCY motif in pink, the putative dimer interface corresponding to EEA1-FYVE dimer region in yellow and the C-terminal RVC motif in green. Additionally, residues on red background represent the residues interacting with Ins(1,3)*P*_2 _(Panel A) and residues interacting with Ins(1,5)*P*_2 _(Panel B).

*At*FYVE domains can be divided into two distinct groups based on different consensus sequences identified via CLUSTALW multiple sequence alignment (Fig. 5). Fig. 5A depicts *At*FYVE domains, which belong to class I-IV. These *Arabidopsis *domains were previously referred to as classic FYVE domains because they contain three classic conserved regions: the N-terminal WxxD, the central R(R/K)HHCR and the C-terminal R(V/I)C motifs [32] implicated in binding the phosphoinositide ligand PtdIns(3)*P*. Class I-IV *At*FYVE proteins have a classic FYVE domain (Fig. 5A) with a conserved motif for PtdIns(3)*P*-binding that is found in FYVE domains of *H. sapiens *[34], *S. cerevisiae, C. elegans *[45] and various other organisms, e.g. *P. troglodytes, M. musculus, R. norvegicus, C. familiaris, B. taurus, G. Gallus*. Class V *At*FYVE domains do not share the N-terminal WxxD motif. Instead they have a WxxG motif, only a G residue or residues that share no similarity to the WxxD or WxxG motifs (Fig. 5). Moreover, the central R(R/K)HHCR motif is replaced by a (K/R)(R/K)HNCY motif, which is atypical and hence the name "variant binding motif" of FYVE domains [32].

We observe that the variable turret loop prior to the R(R/K)HHCR motif, which is associated with membrane penetration of the FYVE domain, and the putative dimerization interface region are made up of residues, which are quite diverse in the various class I-IV FYVE domains. Despite the observed differences in residues, however, all class I-IV *At*FYVE domains share at least one hydrophobic residue within the turret loop and highly hydrophobic dimerization interface regions. AT1G29800-FYVE and AT3G43230-FYVE have an insertion of an additional hydrophobic residue within the turret loop. Class V *At*FYVE domains contain a conserved phenylalanine residue in the second position (with the exception of AT3G47660-FYVE) and a conserved arginine in the last position within the turret loop. As in the case of class I-IV *At*FYVE domains, the putative dimerization interface region of class V *At*FYVE domains is highly hydrophobic. Unlike class I-IV *At*FYVE domains, however, class V *At*FYVE domains dimerization interface regions seems highly conserved with at least three absolutely conserved residues, i.e. AxxAP.

### FYVE domains have the potential to bind headgroups of both PtdIns(3)*P *and PtdIns(5)*P*

**Figure 6:**
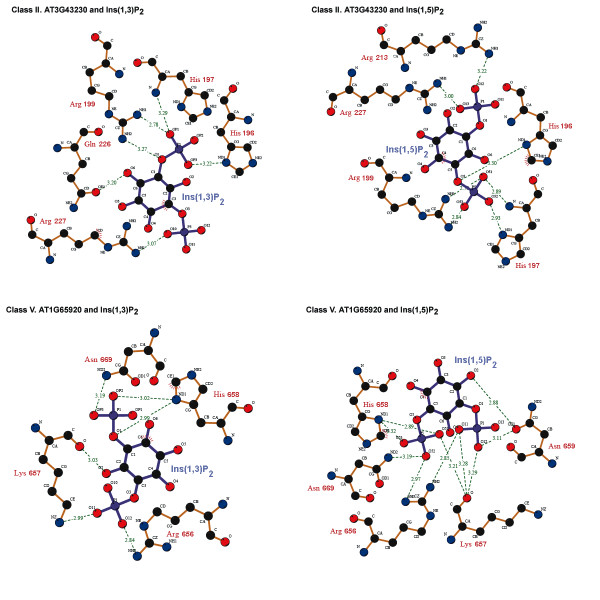
**Ins(1,3)*P*_2 _and Ins(1,5)*P*_2 _coordination**. A schematic representation of the coordination of PtdIns(3)*P *headgroup, Ins(1,3)*P*_2_, and PtdIns(5)*P *headgroup, Ins(1,5)*P*_2_, by the conserved sequence motif of class II *Arabidopsis *AT3G43230-FYVE (classic) and class V *Arabidopsis *AT1G65920-FYVE (variant). The figures were created using LIGPLOT [132].

Preliminary docking studies depicted in Fig. 5 and Fig. 6 show that class I-IV *At*FYVE domains have a potential to bind headgroups of both PtdIns(3)*P *and PtdIns(5)*P *using the same set of residues previously identified to bind the headgroup of PtdIns(3)*P *in other FYVE domains, i.e. the RHHxR motif and the arginine residue of RVC motif (Fig. 5). Class V *At*FYVE domains use the variant signature of residues, i.e. xRKxHNxY motif, and a (L/F/P)YR motif, which overlaps the classic RVC motif, to potentially bind headgroups of PtdIns(3)*P *and PtdIns(5)*P *(Fig. 5B). In addition to the variant residues, our data indicate that a (H/K/N)xx(S/T)(S/N)(K/R)K motif located immediately prior to the dimerization region is also used by class V *At*FYVE domains to recognize either headgroup (Fig. 5B).

## Discussion

Proteins that contain FYVE zinc finger domains have so far been known as effectors of PtdIns(3)*P *playing a major role in endocytic and vesicular trafficking [46-48]. PtdIns(3)*P *is a phosphoinositide that is present at very low levels in plant cells [49-52]. It is synthesized by phosphatidylinositol 3-kinase (PI3K). Both PtdIns(3)*P *and PI3K are essential for normal plant growth [31] and have been implicated in diverse physiological functions, including root nodule formation [53], auxin-induced production of reactive oxygen species (ROS) and root gravitropism [54], root hair curling and *Rhizobium *infection in *M. truncatula *[55], maintenance of the processes essential for root hair cell elongation [56], increased plasma membrane endocytosis and the intracellular production of ROS in the salt tolerance response [57], stomatal closing movement [57,58], and possibly cytokinesis [11]. If we envision plant FYVE domains as being potential effectors of PtdIns(3)*P*, they could play important roles in various physiological processes. In this study we have modeled the structure of all *At*FYVE domains and predicted their membrane targeting behavior based on the biophysical profiles of the modeled structures.

Based on the domain architecture and homology to proteins of known function, we have classified *At*FYVE proteins into five distinct classes (Fig. 2). Similar domain based classifications previously performed for FYVE domain-containing proteins in *H. sapiens*, *C. elegans *and *S. cerevisiae *genomes [34,45] suggested a certain degree of correspondence among the different FYVE proteins in various organisms [34]. However, *At*FYVE proteins are striking in showing no obvious similarities or correspondence to the FYVE proteins included in these domain architecture-based classifications. More specifically, only one class of *At*FYVE proteins corresponds to what was reported in other organisms, i.e. class I in our classification and the corresponding PIKfvye, MmPIKfyve and ScFab1p groups in the other classifications [34,40,45]. Even that correspondence, however, is partial since the *Arabidopsis *counterparts lack the disheveled, Egl-10, and pleckstrin (DEP) domain observed in mammals and worms [34,40,45,59]. The remaining members of *At*FYVE proteins class II-V are unique and exhibit completely different domains suggesting that FYVE domains in plants play a unique role in plant signaling pathways and/or play similar/parallel roles in signaling as other organisms but use different protein players or signaling mechanisms.

The two class I sequences are homologues of the PIKfyve/Fab1 family of phosphatidylinositol phosphate 5-kinases that phosphorylate the D-5 position in phosphatidylinositol (PtdIns) and PtdIns(3)*P *to make PtdIns(5)*P *and PtdIns(3,5)*P*_2_, respectively [60]. PIKfyve/Fab1 proteins bind PtdIns(3)*P *with high specificity through their FYVE domains [15,60] and are known to participate in several aspects of endosomal trafficking functions [61], transduction of osmotic shock signals [62] and other cellular functions in mammals and yeast [40] as well as in plants [63-65]. Recently, the two class I *At*FYVE, PIKfyve proteins, were found to participate in vacuolar rearrangement essential for successful pollen development [63] and our molecular models provide the structural insight into their mode of function. Both members possess the complete classic signature for PtdIns(3)*P-*binding and the conserved hydrophobic motif suggesting that they likely bind membranes using the general mechanism of non-specific electrostatic interactions, followed by membrane penetration of hydrophobic residues close to the PtdIns(3)*P*-binding pocket facilitated by an electrostatic switch coupled with specific interactions with PtdIns(3)*P *as proposed by previous computational modeling studies [22]. These studies have shown that all human FYVE domains have electrostatic equipotential profiles similar to those of Hrs and EEA1 FYVE domains. This electrostatic polarity seems to be characteristic for class I *At*FYVE domains and their *S. cerevisiae *and *C. elegans *homologues (Fig. 4 and Fig. S1 (Supplementary material)). Despite the overall electrostatic profile similarity, AT3G14270-FYVE has a higher net charge (+7 at pH 6.5; Zn ions included) than AT4G33240-FYVE (+3 at pH 6.5; Zn ions included) (Fig 3). Based on the net charge difference, we predict that AT4G33240-FYVE will have a reduced non-specific electrostatic contribution to membrane targeting. Moreover, we predict that its hydrophobic contribution will also be reduced because the conserved hydrophobic motif of AT4G33240-FYVE possesses a valine residue instead of a leucine residue found in AT3G14270-FYVE (Fig. 4). Additionally, FYVE domain dimerization might be important for functional membrane association of AT4G33240-FYVE.

Class II-IV proteins have untouched sequences in terms of functional assignment, which remain annotated as "putative uncharacterized proteins" in various sequence databases. All of them share the complete/nearly complete conserved PtdIns(3)*P*-binding motif and a large basic binding pocket except for class IV AT1G20110-FYVE, which has a significantly reduced basic surface patch in the potential ligand-binding pocket (Fig. 4; class II-IV domains have net charges of +6, +8, +11, and +2, respectively). Class II FYVE domains possess a classic FYVE domain electrostatic profile but their binding signature is missing the first of the arginines in the R(R/K)HHCR motif, which is known to recognize the 1-phosphate of PtdIns(3)*P *headgroup [20]. Even though this residue doesn't participate in the direct recognition of the 3-phosphate, mutational studies suggest that substitution of this arginine substantially reduces the FYVE domain's affinity for PtdIns(3)*P*-containing membranes and potential for membrane localization. The altered signature may slightly reduce the local basic charge in the vicinity of the hydrophobic motif and lower the barrier to membrane penetration. In this class, we predict a classic FYVE domain membrane-targeting behavior with subtle differences that could be verified using mutational studies. Class III FYVE domain on the contrary has the full binding signature and an electrostatic equipotential profile similar to those of Hrs and EEA1 FYVE domains. We predict that this domain will localize to PtdIns(3)*P*-containing membranes using the classic mechanism of action of previously studied FYVE domains with a strong contribution from non-specific electrostatic interactions.

Class IV *At*FYVE domain has the most reduced basic surface patch and the lowest net charge of +2 among *At*FYVE domains. Hydrophobic contribution through membrane insertion will likely be an important component of membrane binding for this class, similar to FENS-FYVE [22], which localizes to endosomal membrane [66] even though it has a weaker positive potential than other known FYVE domains [22].

Class V proteins are the most interesting class of the FYVE domain-containing proteins although much remains to be understood about their function. Out of the 18 human RCC1 superfamily proteins, none corresponds, in their domain architecture to class V FYVE proteins [67]. The closest match, the PAM protein, has 3 RCC1 repeats and a FYVE domain but in a different order and accompanied by domains other than domains found in class V *At*FYVE proteins [67]. In contrast to the traditional seven canonical repeats found is most RCC1-like proteins, there are six RCC1 repeats in some proteins such as WBSCR16, Nek9, RPGR [67] and some *At*FYVE proteins (this study). Since β-propellers (including RCC1 repeats) could be made of a variable number of blades and are thought to evolve by blade duplication and deletion [68], there could be three alternative explanations for the absence of the first canonical RCC1 repeat in some class V *At*FYVE proteins: 1) the second half of blade 1 and the first half of blade 7 engage with one another to form a symmetrical 6-bladed β-propeller; 2) an "open" ring-propeller forms as known for the C-terminal domain of ParC subunit [69] and suggested for the short-form of Alsin [70]; or 3) the first repeat is a non-canonical RCC1 repeat as seen in other proteins [67]. Therefore, despite the sequence differences, it is possible that the 6 RCC1 repeats found in some *At*FYVE adapt a β-propeller structure similar to β-propeller structures found in proteins from other organisms.

Previously, it has been suggested that association with membrane(s) may be crucial for the functioning of this class of AtFYVE proteins given the presence of two phosphoinositide-binding domains, i.e. PH and FYVE domains [32]. Experimental data suggest that class V AT1G65920 PH domain binds to PtdIns(4,5)*P*_2 _while its FYVE domain binds to PtdIns(3)*P *as well as PtdIns(5)*P *[32]. The various members of class V *At*FYVE domains show a high degree of sequence conservation within an enrichment of basic residues throughout the length of the FYVE domain (Fig. 5). The most striking feature of these FYVE domains is the presence of a variant phosphoinositide-binding motif (Fig. 5B), which seems to be unique to plants as is the overall domain architecture of these proteins ([32]; Fig. 5B). When the variant (K/R)(R/K)HNCY motif of class V FYVE domains is used to search for other FYVE domains, only sequences from plants are retrieved, e.g. Q1SA17 (*M. truncatula*), Q1SIN6 (*M. truncatula*), Q84RS2 (*M. sativa*), Q5JL00 (*O. sativa*) Q5N8I7 (*O. sativa*) Q6AV10 (*O. sativa*) Q6L5B2 (*O. sativa*) Q259N3 (*O. sativa*), Q5XWP1 (*S. tuberosum*), Q60CZ5 (*S. tuberosum*), Q60CZ5 (*S. demissum*) or Q5EWZ4 (*T. turgidum*).

The obvious question that comes to mind is whether this variant signature is responsible for an altered binding specificity in this class of FYVE proteins and therefore associated with a novel pattern of membrane/sub-cellular targeting. Within mammalian cells, FYVE domains are highly conserved and seem to select PtdIns(3)*P *over other phosphoinositides [16]. Despite the conservation in the overall mechanism, there are significant differences in the specificity and affinity of individual FYVE domains towards phosphoinositides. In fact, EEA1 has affinity for PtdIns(5)*P *as well, perhaps because PtdIns(3)*P *and PtdIns(5)*P *are similar in all aspects except having the phosphomonoester in a different position [38]. Consequently, PtdIns(5)*P *has been shown to induce small but important chemical shift changes similar to those induced by PtdIns(3)*P *in the binding motif residues with the exception of one arginine, which remains practically unaltered by PtdIns(5)*P *[38]. PtdIns(3)*P *specific recognition by the FYVE domain seems to involve indirect recognition of this specific ligand by exclusion of alternatively phosphorylated phosphoinositides: the two residues implicated in this are the aspartic acid of the N-terminal WxxD motif and the second histidine of the central HHCR motif [20]. Both of these motifs are substituted in class V variant *At*FYVE domains (Fig. 5) by the WxxG and HNCY motifs, respectively. This opens up the possibility that class V FYVE domains may have the potential to interact equally or better with phosphoinositide ligands other than PtdIns(3)*P*. Our preliminary docking analysis of classic as well as variant motif-containing *At*FYVE domains seem to suggest that both have the potential to interact with PtdIns(3)*P *and PtdIns(5)*P *headgroups using practically the same set of residues (Fig. 5). Additionally, our analysis reveals a highly conserved putative ligand-association motif located immediately prior to the dimerization region present only within the class V proteins (Fig. 5). Class V *At*FYVE domains are also different in exhibiting very large basic surface patches with prominent hydrophobic motifs. These patches are the largest observed among FYVE domains classified to date [71,72]. We predict that class V *At*FYVE domains target to the membrane with highly significant contributions from non-specific electrostatics and hydrophobic interactions, coupled with specific interactions with PtdIns(3)*P *and/or PtdIns(5)*P *using the variant binding residues and an additional conserved motif specific to this class of FYVE domains.

Based on experimental studies, it has been suggested that the strength of the positive potential and the identity of the hydrophobic residues near the binding site may be two key factors, which are critical in determining which FYVE domains act alone, undergo dimerization or require additional partners before anchoring to the membrane [22]. For example, SARA-FYVE was predicted and verified experimentally to associate with the membrane with significant contributions from non-specific electrostatic and hydrophobic interactions given its net charge of +12 (zinc ions included) as well as the presence of phenylalanine at the conserved hydrophobic position [22,71,73]. Our data suggests that *At*FYVE domains engage in both non-specific electrostatic and PtdIns(3)*P*-induced hydrophobic interactions for membrane localization, the contribution differing for individual domains as described earlier. Additionally, dimerization may play an important role in the membrane recruitment of FYVE domains [21,74] and it appears that the free energy contributions to the membrane association are additive for each monomer of the EEA1-FYVE dimer [22]. The dimer interface regions of *At*FYVE domains are longer and more hydrophobic (Fig. 5) than the equivalent region of EEA1-FYVE and predicted region of SARA-FYVE [72,75] suggesting that all *At*FYVE domains have the potential to dimerize and associate with membrane(s) as dimers.

### Conclusions

Overall, *At*FYVE proteins are quite distinct from other organisms, exhibiting unique domain architectures, biophysical properties as well as altered binding motifs. The biophysical profiles of the modeled FYVE domains in *Arabidopsis *suggest membrane-targeting mechanisms ranging from the previously described classic modes to the novel binding mode of the class V FYVE domains, which seem to be found only in plants. Our predictions provide a foundation for designing directed mutational studies to confirm these behaviors, which is crucial to the understanding of the role of these domains in important plant signaling pathways, something that has so far not been explored.

## Methods

### Arabidopsis FYVE proteins

The accession numbers of the *At*FYVE proteins were identified using a computational pipeline for automated high-throughput modeling [36], which run against *Arabidopsis *protein sequence database (TAIR6_pep_20051108). The *At*FYVE protein sequences corresponding to the identified accession numbers were retrieved from KEGG GENES [76,77] and verified for presence of FYVE domain with SMART [78-80].

### Sequence verification

To verify the total number and individual accession numbers of *At*FYVE proteins obtained with pipeline, three additional methods were employed: 1) search of publicly available sequence databases: Swiss-Prot/TrEMBL [81,82], NCBI [83,84] and UniProt [85-87]; 2) query performed by the *Arabidopsis *Information Resource (TAIR) for BLASTn 2.2.14 [88]; and 3) MOTIF search [77] using a manually derived pattern specific to *At*FYVE domains in PROSITE format offered by GenomeNet service [77].

### Domain architecture analyses

Each *At*FYVE protein sequence was analyzed by searching against Pfam [89], SMART [78-80], Conserved Domain Database v2.10 and CD-Search [90-94] and Clusters of Orthologous Groups [95,96]. All sequences were then subgrouped according to consensus domain architecture.

### Modeling methodology

There is no single homology modeling program/routine that has been singled out as the best method for comparative modeling [97]. To generate high-quality models for the *At*FYVE domains, we implemented a number of programs to create many different alternative alignments and models followed by a quality assessment and a selection process. We used two separate approaches: automated and manual. The automated approach involved the use of a high-throughput computational pipeline, which uses its own built in alignment, modeling and evaluation methods [36] as well as Pudge for modeling and evaluation [98]. The manual approach is based on choosing several alternative options for each step in the process of creating the homology models as previously detailed by Singh and Murray [99]. The scheme involves the use of multiple approaches at each step: 1) choice of a suitable structural template, 2) alignment of the template and target sequences, 3) model building, and 4) model evaluation and refinement using 3D-JIGSAW [100-102], Modeller 8v1 [103,104], NEST [105], LOOPP [106-108], HOMER [109], CPH [110], PHYRE [111], manual editing using GeneDoc [112], guided by Verify3 D [113,114] and Prosa [115]. Loop refinement and side chain conformations were performed using individual modeling programs whenever available. In addition, loop refinement was done with Loopy [116] and the prediction of side-chain conformations with SCWRL3.0 [117] and SCAP [118-120].

### Analysis of the models

The models were analyzed for their sequence, structural and biophysical properties. The analyses of biophysical properties including the electrostatics, hydrophobicity and shape of each model were conducted using the surface property analysis tools in the program GRASP [121]. The pKa values of ionizable amino acid side chains in *At*FYVE domains as well as total charges were computed using the automated system H++ [122-124], which is based on solutions to the Poisson-Boltzmann equation. The calculations were performed using default settings. The reported total charges was calculated at pH 6.5 because EEA1-FYVE was estimated to exist in bound state at low pH of 6.0-6.6 and only half of the protein was estimated to remain active at the cytostolic pH of 7.3 [20].

### Ligand preparation

Ins(1,3)*P*_2_, Ins(1,4)*P*_2_, Ins(1,4,5)*P*_3_, Ins(1,3,5)*P*_3_, Ins(1,3,4)*P*_3_, and Ins(1,3,4,5)*P*_4 _ligands were extracted from their corresponding PDB files. Ins(1,5)*P*_2 _ligands were created from Ins(1,4,5)*P*_3 _ligands in Chimera [125] and energy minimized. Hydrogens were added to all ligands using Chimera [125]. Gasteiger charges were calculated for all ligands.

### Phosphoinositides docking and analysis of resulting interactions

Rigid and flexible docking was performed using DOCK 6.1 [126] and DOCK 6.1 suite programs. A molecular surface of the receptor was created with DMS [127,128]. Spheres were generated with Sphgen_cpp v1.2, which was modified by Andrew Magis from its original version called Sphgen [126]. The resulting file was edited to include only spheres grouped within the first cluster. Grids were generated with GRID [129,130]. Contact scores and energy scores were calculated using an energy cutoff distance of 5.0 A. Our docking technique was validated by docking Ins(1,3)*P*_2 _of known FYVE domains into their corresponding solved structures. Although FYVE domains are suggested to bind only Ins(1,3)*P*_2 _and Ins(1,5)*P*_2_, we also docked Ins(1,4)*P*_2_, Ins(1,4,5)*P*_3_, Ins(1,3,5)*P*_3_, Ins(1,3,4)*P*_3_, and Ins(1,3,4,5)*P*_4 _as controls. Following the initial validation we used our approach to dock three Ins(1,3)*P*_2 _and three Ins(1,5)*P*_2 _ligands using rigid and flexible docking scenarios with the predictive models of *At*FYVE domains. In the end, each predictive model was subjected to twelve docking runs, six for each headgroup. A given residue is reported to interact with the headgroup only if it does so 50% or more of the time (i.e. 3 or more times) as evaluated by the Ligand-Protein Contacts (LPC) server [131].

### Electronic supplementary material

The sequences and coordinate files representing our models for all *At*FYVE domains as well as other supplementary information (GRASP images and structure verification plots, and alignment files) are available at the following website: http://userhome.brooklyn.cuny.edu/ssingh/arabidopsis/FYVE/fyve.html.

## List of Abbreviations and symbols

*AT*: *Arabidopsis thaliana*; ATS1: Alpha Tubulin Suppressor 1; DUF500: Domain of Unknown Function 500; EEA1: Early Endosomal Antigen 1; FAB1P: Formation of aploid and binucleate cells; FCP: Fucoxanthin Chlorophyll a/c-Binding; FYVE: Fab1, YOTB, Vac1, and EEA1; HRS: Hepatocyte growth factor-regulated tyrosine kinase substrate; MVB: MultiVesicular Bodies; PH_PLC: Pleckstrin Homology of Phospholipase C; PIKFYVE: PhosphatidylInositol 3-phosphate 5-Kinase; PI 3-kinase: PhosphoInositide 3-Kinase; PRAF: PH, RCC1 and FYVE; PTDINS: PhosphatIdylinositol; PTDINS(3)*P*: PhosphatIdylinositol 3 Phosphate; PtdIns(5)*P*: PhosphatIdylinositol 5 Phosphate; RCC1: Regulator of Chromosome Condensation 1; SARA: Smad Anchor for Receptor Activation; VPS27P: Vacuolar protein sorting mutant 27 phenotype; VPS34P: Vacuolar protein sorting mutant 34; ROS: Reactive Oxygen Species.

## Authors' contributions

EW carried out the modeling and sequence/structure analyses and drafted the manuscript. SMS conceived of the study, participated in its design and coordination, guided the analyses and refined the manuscript. All authors read and approved the final manuscript.

## References

[B1] Stenmark H Aasland R Toh BH D'Arrigo A Endosomal localization of the autoantigen EEA1 is mediated by a zinc-binding FYVE finger J Biol Chem 1996 271 39 24048 24054 10.1074/jbc.271.39.24048 8798641

[B2] Petiot A Faure J Stenmark H Gruenberg J PI3P signaling regulates receptor sorting but not transport in the endosomal pathway J Cell Biol 2003 162 6 971 979 10.1083/jcb.200303018 12975344 PMC2172844

[B3] Wurmser AE Gary JD Emr SD Phosphoinositide 3-Kinases and Their FYVE Domain-containing Effectors as Regulators of Vacuolar/Lysosomal Membrane Trafficking Pathways J Biol Chem 1999 274 14 9129 9132 10.1074/jbc.274.14.9129 10092582

[B4] Simonsen A Lippe R Christoforidis S Gaullier JM Brech A Callaghan J Toh BH Murphy C Zerial M Stenmark H EEA1 links PI(3)K function to Rab5 regulation of endosome fusion Nature 1998 394 6692 494 498 10.1038/28879 9697774

[B5] Rutherford AC Traer C Wassmer T Pattni K Bujny MV Carlton JG Stenmark H Cullen PJ The mammalian phosphatidylinositol 3-phosphate 5-kinase (PIKfyve) regulates endosome-to-TGN retrograde transport J Cell Sci 2006 119 Pt 19 3944 3957 10.1242/jcs.03153 16954148 PMC1904490

[B6] Estrada L Caron E Gorski JL Fgd1, the Cdc42 guanine nucleotide exchange factor responsible for faciogenital dysplasia, is localized to the subcortical actin cytoskeleton and Golgi membrane Hum Mol Genet 2001 10 5 485 495 10.1093/hmg/10.5.485 11181572

[B7] Cooke FT Dove SK McEwen RK Painter G Holmes AB Hall MN Michell RH Parker PJ The stress-activated phosphatidylinositol 3-phosphate 5-kinase Fab1p is essential for vacuole function in S. cerevisiae Curr Biol 1998 8 22 1219 1222 10.1016/S0960-9822(07)00513-1 9811604

[B8] Gary JD Wurmser AE Bonangelino CJ Weisman LS Emr SD Fab1p is essential for PtdIns(3)P 5-kinase activity and the maintenance of vacuolar size and membrane homeostasis J Cell Biol 1998 143 1 65 79 10.1083/jcb.143.1.65 9763421 PMC2132800

[B9] Odorizzi G Babst M Emr SD Fab1p PtdIns(3)P 5-kinase function essential for protein sorting in the multivesicular body Cell 1998 95 6 847 858 10.1016/S0092-8674(00)81707-9 9865702

[B10] Tsukazaki T Chiang TA Davison AF Attisano L Wrana JL SARA, a FYVE domain protein that recruits Smad2 to the TGFbeta receptor Cell 1998 95 6 779 791 10.1016/S0092-8674(00)81701-8 9865696

[B11] Seet LF Hong W Endofin, an endosomal FYVE domain protein J Biol Chem 2001 276 45 42445 42454 10.1074/jbc.M105917200 11546807

[B12] Seet LF Hong W Endofin recruits clathrin to early endosomes via TOM1 J Cell Sci 2005 118 Pt 3 575 587 10.1242/jcs.01628 15657082

[B13] Seet LF Liu N Hanson BJ Hong W Endofin recruits TOM1 to endosomes J Biol Chem 2004 279 6 4670 4679 10.1074/jbc.M311228200 14613930

[B14] Misra S Hurley JH Crystal structure of a phosphatidylinositol 3-phosphate-specific membrane-targeting motif, the FYVE domain of Vps27p Cell 1999 97 5 657 666 10.1016/S0092-8674(00)80776-X 10367894

[B15] Burd CG Emr SD Phosphatidylinositol(3)-phosphate signaling mediated by specific binding to RING FYVE domains Mol Cell 1998 2 1 157 162 10.1016/S1097-2765(00)80125-2 9702203

[B16] Gaullier JM Simonsen A D'Arrigo A Bremnes B Stenmark H Aasland R FYVE fingers bind PtdIns(3)P Nature 1998 394 6692 432 433 10.1038/28767 9697764

[B17] Patki V Lawe DC Corvera S Virbasius JV Chawla A A functional PtdIns(3)P-binding motif Nature 1998 394 6692 433 434 10.1038/28771 9697765

[B18] Patki V Virbasius J Lane WS Toh BH Shpetner HS Corvera S Identification of an early endosomal protein regulated by phosphatidylinositol 3-kinase Proc Natl Acad Sci USA 1997 94 14 7326 7330 10.1073/pnas.94.14.7326 9207090 PMC23820

[B19] Gaullier JM Ronning E Gillooly DJ Stenmark H Interaction of the EEA1 FYVE finger with phosphatidylinositol 3-phosphate and early endosomes. Role of conserved residues J Biol Chem 2000 275 32 24595 24600 10.1074/jbc.M906554199 10807926

[B20] Kutateladze TG Phosphatidylinositol 3-phosphate recognition and membrane docking by the FYVE domain Biochim Biophys Acta 2006 1761 8 868 877 16644267 10.1016/j.bbalip.2006.03.011 PMC2740714

[B21] Kutateladze TG Ogburn KD Watson WT de Beer T Emr SD Burd CG Overduin M Phosphatidylinositol 3-phosphate recognition by the FYVE domain Mol Cell 1999 3 6 805 811 10.1016/S1097-2765(01)80013-7 10394369

[B22] Diraviyam K Stahelin RV Cho W Murray D Computer modeling of the membrane interaction of FYVE domains J Mol Biol 2003 328 3 721 736 10.1016/S0022-2836(03)00325-5 12706728

[B23] Kutateladze TG Capelluto DG Ferguson CG Cheever ML Kutateladze AG Prestwich GD Overduin M Multivalent mechanism of membrane insertion by the FYVE domain J Biol Chem 2004 279 4 3050 3057 10.1074/jbc.M309007200 14578346

[B24] Stahelin RV Long F Diraviyam K Bruzik KS Murray D Cho W Phosphatidylinositol 3-phosphate induces the membrane penetration of the FYVE domains of Vps27p and Hrs J Biol Chem 2002 277 29 26379 26388 10.1074/jbc.M201106200 12006563

[B25] Kutateladze T Overduin M Structural mechanism of endosome docking by the FYVE domain Science 2001 291 5509 1793 1796 10.1126/science.291.5509.1793 11230696

[B26] Mao Y Nickitenko A Duan X Lloyd TE Wu MN Bellen H Quiocho FA Crystal structure of the VHS and FYVE tandem domains of Hrs, a protein involved in membrane trafficking and signal transduction Cell 2000 100 4 447 456 10.1016/S0092-8674(00)80680-7 10693761

[B27] Hayakawa A Hayes SJ Lawe DC Sudharshan E Tuft R Fogarty K Lambright D Corvera S Structural basis for endosomal targeting by FYVE domains J Biol Chem 2004 279 7 5958 5966 10.1074/jbc.M310503200 14594806

[B28] He J Vora M Haney RM Filonov GS Musselman CA Burd CG Kutateladze AG Verkhusha VV Stahelin RV Kutateladze TG Membrane insertion of the FYVE domain is modulated by pH Proteins 2009 76 4 852 860 10.1002/prot.22392 19296456 PMC2909462

[B29] Psachoulia E Sansom MS PX- and FYVE-mediated interactions with membranes: simulation studies Biochemistry 2009 48 23 5090 5095 10.1021/bi900435m 19408958

[B30] Kim DH Eu YJ Yoo CM Kim YW Pih KT Jin JB Kim SJ Stenmark H Hwang I Trafficking of phosphatidylinositol 3-phosphate from the trans-Golgi network to the lumen of the central vacuole in plant cells Plant Cell 2001 13 2 287 301 10.1105/tpc.13.2.287 11226186 PMC102243

[B31] Welters P Takegawa K Emr SD Chrispeels MJ AtVPS34, a Phosphatidylinositol 3-Kinase of Arabidopsis thaliana, is an Essential Protein with Homology to a Calcium-Dependent Lipid Binding Domain PNAS 1994 91 24 11398 11402 10.1073/pnas.91.24.11398 7972072 PMC45238

[B32] Jensen RB La Cour T Albrethsen J Nielsen M Skriver K FYVE zinc-finger proteins in the plant model Arabidopsis thaliana: identification of PtdIns3P-binding residues by comparison of classic and variant FYVE domains Biochem J 2001 359 Pt 1 165 173 10.1042/0264-6021:3590165 11563980 PMC1222132

[B33] Heras B Drobak BK PARF-1: an Arabidopsis thaliana FYVE-domain protein displaying a novel eukaryotic domain structure and phosphoinositide affinity J Exp Bot 2002 53 368 565 567 10.1093/jexbot/53.368.565 11847256

[B34] Stenmark H Aasland R Driscoll PC The phosphatidylinositol 3-phosphate-binding FYVE finger FEBS Lett 2002 513 1 77 84 10.1016/S0014-5793(01)03308-7 11911884

[B35] AGI Analysis of the genome sequence of the flowering plant Arabidopsis thaliana Nature 2000 408 6814 796 815 10.1038/35048692 11130711

[B36] Mirkovic N Li Z Parnassa A Murray D Strategies for high-throughput comparative modeling: Applications to leverage analysis in structural genomics and protein family organization Proteins 2006 66 4 766 777 10.1002/prot.21191 17154423

[B37] Dumas JJ Merithew E Sudharshan E Rajamani D Hayes S Lawe D Corvera S Lambright DG Multivalent endosome targeting by homodimeric EEA1 Mol Cell 2001 8 5 947 958 10.1016/S1097-2765(01)00385-9 11741531

[B38] Drobak BK Heras B Nuclear phosphoinositides could bring FYVE alive Trends Plant Sci 2002 7 3 132 138 10.1016/S1360-1385(01)02213-0 11906837

[B39] van Leeuwen W Okresz L Bogre L Munnik T Learning the lipid language of plant signalling Trends Plant Sci 2004 9 8 378 384 10.1016/j.tplants.2004.06.008 15358268

[B40] Cooke FT Phosphatidylinositol 3,5-bisphosphate: metabolism and function Arch Biochem Biophys 2002 407 2 143 151 10.1016/S0003-9861(02)00487-3 12413484

[B41] Mueller-Roeber B Pical C Inositol phospholipid metabolism in Arabidopsis. Characterized and putative isoforms of inositol phospholipid kinase and phosphoinositide-specific phospholipase C Plant Physiol 2002 130 1 22 46 10.1104/pp.004770 12226484 PMC166537

[B42] Otomo A Hadano S Okada T Mizumura H Kunita R Nishijima H Showguchi-Miyata J Yanagisawa Y Kohiki E Suga E ALS2, a novel guanine nucleotide exchange factor for the small GTPase Rab5, is implicated in endosomal dynamics Hum Mol Genet 2003 12 14 1671 1687 10.1093/hmg/ddg184 12837691

[B43] Rosa JL Casaroli-Marano RP Buckler AJ Vilaro S Barbacid M p619, a giant protein related to the chromosome condensation regulator RCC1, stimulates guanine nucleotide exchange on ARF1 and Rab proteins Embo J 1996 15 16 4262 4273 8861955 PMC452152

[B44] Renault L Nassar N Vetter I Becker J Klebe C Roth M Wittinghofer A The 1.7 A crystal structure of the regulator of chromosome condensation (RCC1) reveals a seven-bladed propeller Nature 1998 392 6671 97 101 10.1038/32204 9510255

[B45] Stenmark H Aasland R FYVE-finger proteins--effectors of an inositol lipid J Cell Sci 1999 112 Pt 23 4175 4183 10564636 10.1242/jcs.112.23.4175

[B46] Corvera S D'Arrigo A Stenmark H Phosphoinositides in membrane traffic Curr Opin Cell Biol 1999 11 4 460 465 10.1016/S0955-0674(99)80066-0 10449332

[B47] Simonsen A Wurmser AE Emr SD Stenmark H The role of phosphoinositides in membrane transport Curr Opin Cell Biol 2001 13 4 485 492 10.1016/S0955-0674(00)00240-4 11454456

[B48] Stenmark H Gillooly DJ Intracellular trafficking and turnover of phosphatidylinositol 3-phosphate Semin Cell Dev Biol 2001 12 2 193 199 10.1006/scdb.2000.0236 11292385

[B49] Meijer HJG Berrie CP Lurisci C Divecha N Musgrave A Munnik T Identification of a new polyphosphoinositide in plants, phosphatidylinositol 5-phosphate and its accumulation upon osmotic stress Biochem J 2001 360 491 498 10.1042/0264-6021:3600491 11716778 PMC1222250

[B50] Brearley CA Hanke DE 3- and 4-phosphorylated phosphatidylinositols in the aquatic plant Spirodela polyrhiza L Biochem J 1992 283 Pt 1 255 260 1567374 10.1042/bj2830255 PMC1131022

[B51] Munnik T Irvine RF Musgrave A Rapid turnover of phosphatidylinositol 3-phosphate in the green alga Chlamydomonas eugametos: signs of a phosphatidylinositide 3-kinase signalling pathway in lower plants? Biochem J 1994 298 Pt 2 269 273 8135730 10.1042/bj2980269 PMC1137935

[B52] Munnik T Musgrave A De Vrije T Rapid turnover of polyphosphoinositides in carnation flower petals Planta 1994 193 89 98 10.1007/BF00191611

[B53] Hong Z Verma DP A phosphatidylinositol 3-kinase is induced during soybean nodule organogenesis and is associated with membrane proliferation Proc Natl Acad Sci USA 1994 91 20 9617 9621 10.1073/pnas.91.20.9617 7937816 PMC44864

[B54] Joo JH Yoo HJ Hwang I Lee JS Nam KH Bae YS Auxin-induced reactive oxygen species production requires the activation of phosphatidylinositol 3-kinase FEBS Lett 2005 579 5 1243 1248 10.1016/j.febslet.2005.01.018 15710420

[B55] Peleg-Grossman S Volpin H Levine A Root hair curling and Rhizobium infection in Medicago truncatula are mediated by phosphatidylinositide-regulated endocytosis and reactive oxygen species J Exp Bot 2007 58 7 1637 1649 10.1093/jxb/erm013 17420174

[B56] Lee Y Bak G Choi Y Chuang WI Cho HT Lee Y Roles of phosphatidylinositol 3-kinase in root hair growth Plant Physiol 2008 147 2 624 635 10.1104/pp.108.117341 18408046 PMC2409009

[B57] Leshem Y Seri L Levine A Induction of phosphatidylinositol 3-kinase-mediated endocytosis by salt stress leads to intracellular production of reactive oxygen species and salt tolerance Plant J 2007 51 2 185 197 10.1111/j.1365-313X.2007.03134.x 17521408

[B58] Jung JY Kim YW Kwak JM Hwang JU Young J Schroeder JI Hwang I Lee Y Phosphatidylinositol 3- and 4-phosphate are required for normal stomatal movements Plant Cell 2002 14 10 2399 2412 10.1105/tpc.004143 12368494 PMC151225

[B59] Shisheva A PIKfyve: the road to PtdIns 5-P and PtdIns 3,5-P(2) Cell Biol Int 2001 25 12 1201 1206 10.1006/cbir.2001.0803 11748912

[B60] Sbrissa D Ikonomov OC Shisheva A Phosphatidylinositol 3-phosphate-interacting domains in PIKfyve. Binding specificity and role in PIKfyve. Endomenbrane localization J Biol Chem 2002 277 8 6073 6079 10.1074/jbc.M110194200 11706043

[B61] Shisheva A PIKfyve: Partners, significance, debates and paradoxes Cell Biol Int 2008 32 6 591 604 10.1016/j.cellbi.2008.01.006 18304842 PMC2491398

[B62] Dove SK Cooke FT Douglas MR Sayers LG Parker PJ Michell RH Osmotic stress activates phosphatidylinositol-3,5-bisphosphate synthesis Nature 1997 390 6656 187 192 10.1038/36613 9367158

[B63] Whitley P Hinz S Doughty J Arabidopsis FAB1/PIKfyve proteins are essential for development of viable pollen Plant Physiol 2009 151 4 1812 1822 10.1104/pp.109.146159 19846542 PMC2785992

[B64] Zonia L Munnik T Osmotically induced cell swelling versus cell shrinking elicits specific changes in phospholipid signals in tobacco pollen tubes Plant Physiol 2004 134 2 813 823 10.1104/pp.103.029454 14739344 PMC344556

[B65] Meijer HJG Divecha N van den Ende H Musgrave A Munnik T Hyperosmotic stress induces rapid synthesis of phosphatidyl-D-inositol 3,5-bisphosphate in plant cells Planta 1999 208 294 298 10.1007/s004250050561

[B66] Ridley SH Ktistakis N Davidson K Anderson KE Manifava M Ellson CD Lipp P Bootman M Coadwell J Nazarian A FENS-1 and DFCP1 are FYVE domain-containing proteins with distinct functions in the endosomal and Golgi compartments J Cell Sci 2001 114 Pt 22 3991 4000 11739631 10.1242/jcs.114.22.3991

[B67] Hadjebi O Casas-Terradellas E Garcia-Gonzalo FR Rosa JL The RCC1 superfamily: from genes, to function, to disease Biochim Biophys Acta 2008 1783 8 1467 1479 10.1016/j.bbamcr.2008.03.015 18442486

[B68] Chaudhuri I Soding J Lupas AN Evolution of the beta-propeller fold Proteins 2008 71 2 795 803 10.1002/prot.21764 17979191

[B69] Hsieh TJ Farh L Huang WM Chan NL Structure of the topoisomerase IV C-terminal domain: a broken beta-propeller implies a role as geometry facilitator in catalysis J Biol Chem 2004 279 53 55587 55593 10.1074/jbc.M408934200 15466871

[B70] Soares DC Barlow PN Porteous DJ Devon RS An interrupted beta-propeller and protein disorder: structural bioinformatics insights into the N-terminus of alsin Journal of molecular modeling 2009 15 2 113 122 10.1007/s00894-008-0381-1 19023603

[B71] Itoh F Divecha N Brocks L Oomen L Janssen H Calafat J Itoh S Dijke Pt P The FYVE domain in Smad anchor for receptor activation (SARA) is sufficient for localization of SARA in early endosomes and regulates TGF-beta/Smad signalling Genes Cells 2002 7 3 321 331 10.1046/j.1365-2443.2002.00519.x 11918675

[B72] Qin BY Lam SS Correia JJ Lin K Smad3 allostery links TGF-beta receptor kinase activation to transcriptional control Genes Dev 2002 16 15 1950 1963 10.1101/gad.1002002 12154125 PMC186427

[B73] Panopoulou E Gillooly DJ Wrana JL Zerial M Stenmark H Murphy C Fotsis T Early endosomal regulation of Smad-dependent signaling in endothelial cells J Biol Chem 2002 277 20 18046 18052 10.1074/jbc.M107983200 11877415

[B74] Callaghan J Simonsen A Gaullier JM Toh BH Stenmark H The endosome fusion regulator early-endosomal autoantigen 1 (EEA1) is a dimer Biochem J 1999 338 Pt 2 539 543 10.1042/0264-6021:3380539 10024533 PMC1220083

[B75] Blatner NR Stahelin RV Diraviyam K Hawkins PT Hong W Murray D Cho W The molecular basis of the differential subcellular localization of FYVE domains J Biol Chem 2004 279 51 53818 53827 10.1074/jbc.M408408200 15452113

[B76] Kanehisa M The KEGG database Novartis Found Symp 2002 247 91 101 discussion 101-103, 119-128, 244-152.full_text 12539951

[B77] Kanehisa M Goto S Hattori M Aoki-Kinoshita KF Itoh M Kawashima S Katayama T Araki M Hirakawa M From genomics to chemical genomics: new developments in KEGG Nucl Acids Res 2006 34 suppl_1 D354 357 10.1093/nar/gkj102 16381885 PMC1347464

[B78] Letunic I Copley RR Pils B Pinkert S Schultz J Bork P SMART 5: domains in the context of genomes and networks Nucleic Acids Res 2006 34 Database D257 260 10.1093/nar/gkj079 16381859 PMC1347442

[B79] Schultz J Copley RR Doerks T Ponting CP Bork P SMART: a web-based tool for the study of genetically mobile domains Nucleic Acids Res 2000 28 1 231 234 10.1093/nar/28.1.231 10592234 PMC102444

[B80] Schultz J Milpetz F Bork P Ponting CP SMART, a simple modular architecture research tool: Identification of signaling domains PNAS 1998 95 11 5857 5864 10.1073/pnas.95.11.5857 9600884 PMC34487

[B81] Bairoch A Boeckmann B The SWISS-PROT protein sequence data bank: current status Nucleic Acids Res 1994 22 17 3578 3580 10.1093/nar/22.17.3626 7937062 PMC308324

[B82] Boeckmann B Blatter MC Famiglietti L Hinz U Lane L Roechert B Bairoch A Protein variety and functional diversity: Swiss-Prot annotation in its biological context C R Biol 2005 328 10-11 882 899 10.1016/j.crvi.2005.06.001 16286078

[B83] Benson DA Karsch-Mizrachi I Lipman DJ Ostell J Wheeler DL GenBank Nucleic Acids Res 2006 34 Database D16 20 10.1093/nar/gkj157 16381837 PMC1347519

[B84] Wheeler DL Barrett T Benson DA Bryant SH Canese K Chetvernin V Church DM DiCuccio M Edgar R Federhen S Database resources of the National Center for Biotechnology Information Nucleic Acids Res 2006 34 Database D173 180 10.1093/nar/gkj158 16381840 PMC1347520

[B85] Apweiler R Bairoch A Wu CH Barker WC Boeckmann B Ferro S Gasteiger E Huang H Lopez R Magrane M UniProt: the Universal Protein knowledgebase Nucleic Acids Res 2004 32 Database D115 119 10.1093/nar/gkh131 14681372 PMC308865

[B86] Bairoch A Apweiler R Wu CH Barker WC Boeckmann B Ferro S Gasteiger E Huang H Lopez R Magrane M The Universal Protein Resource (UniProt) Nucleic Acids Res 2005 33 Database D154 159 15608167 10.1093/nar/gki070 PMC540024

[B87] Wu CH Apweiler R Bairoch A Natale DA Barker WC Boeckmann B Ferro S Gasteiger E Huang H Lopez R The Universal Protein Resource (UniProt): an expanding universe of protein information Nucleic Acids Res 2006 34 Database D187 191 10.1093/nar/gkj161 16381842 PMC1347523

[B88] Altschul SF Madden TL Schaffer AA Zhang J Zhang Z Miller W Lipman DJ Gapped BLAST and PSI-BLAST: a new generation of protein database search programs Nucleic Acids Res 1997 25 17 3389 3402 10.1093/nar/25.17.3389 9254694 PMC146917

[B89] Bateman A Coin L Durbin R Finn RD Hollich V Griffiths-Jones S Khanna A Marshall M Moxon S Sonnhammer EL The Pfam protein families database Nucleic Acids Res 2004 32 Database D138 141 10.1093/nar/gkh121 14681378 PMC308855

[B90] Marchler-Bauer A Anderson JB Cherukuri PF DeWeese-Scott C Geer LY Gwadz M He S Hurwitz DI Jackson JD Ke Z CDD: a Conserved Domain Database for protein classification Nucleic Acids Res 2005 33 Database D192 196 15608175 10.1093/nar/gki069 PMC540023

[B91] Marchler-Bauer A Anderson JB Derbyshire MK DeWeese-Scott C Gonzales NR Gwadz M Hao L He S Hurwitz DI Jackson JD CDD: a conserved domain database for interactive domain family analysis Nucleic Acids Res 2007 35 Database D237 240 10.1093/nar/gkl951 17135202 PMC1751546

[B92] Marchler-Bauer A Anderson JB DeWeese-Scott C Fedorova ND Geer LY He S Hurwitz DI Jackson JD Jacobs AR Lanczycki CJ CDD: a curated Entrez database of conserved domain alignments Nucleic Acids Res 2003 31 1 383 387 10.1093/nar/gkg087 12520028 PMC165534

[B93] Marchler-Bauer A Bryant SH CD-Search: protein domain annotations on the fly Nucleic Acids Res 2004 32 Web Server W327 331 10.1093/nar/gkh454 15215404 PMC441592

[B94] Marchler-Bauer A Panchenko AR Shoemaker BA Thiessen PA Geer LY Bryant SH CDD: a database of conserved domain alignments with links to domain three-dimensional structure Nucleic Acids Res 2002 30 1 281 283 10.1093/nar/30.1.281 11752315 PMC99109

[B95] Tatusov RL Fedorova ND Jackson JD Jacobs AR Kiryutin B Koonin EV Krylov DM Mazumder R Mekhedov SL Nikolskaya AN The COG database: an updated version includes eukaryotes BMC Bioinformatics 2003 4 41 10.1186/1471-2105-4-41 12969510 PMC222959

[B96] Tatusov RL Koonin EV Lipman DJ A genomic perspective on protein families Science 1997 278 5338 631 637 10.1126/science.278.5338.631 9381173

[B97] Wallner B Elofsson A All are not equal: A benchmark of different homology modeling programs Protein Sci 2005 14 5 1315 1327 10.1110/ps.041253405 15840834 PMC2253266

[B98] Petrey D Honig B Protein structure prediction: inroads to biology Mol Cell 2005 20 6 811 819 10.1016/j.molcel.2005.12.005 16364908

[B99] Singh SM Murray D Molecular modeling of the membrane targeting of phospholipase C pleckstrin homology domains Protein Sci 2003 12 9 1934 1953 10.1110/ps.0358803 12930993 PMC2323991

[B100] Bates PA Kelley LA MacCallum RM Sternberg MJ Enhancement of protein modeling by human intervention in applying the automatic programs 3D-JIGSAW and 3D-PSSM Proteins 2001 Suppl 5 39 46 10.1002/prot.1168 11835480

[B101] Bates PA Sternberg MJ Model building by comparison at CASP3: using expert knowledge and computer automation Proteins 1999 Suppl 3 47 54 10.1002/(SICI)1097-0134(1999)37:3+<47::AID-PROT7>3.0.CO;2-F 10526351

[B102] Contreras-Moreira B Bates PA Domain fishing: a first step in protein comparative modelling Bioinformatics 2002 18 8 1141 1142 10.1093/bioinformatics/18.8.1141 12176841

[B103] Marti-Renom MA Stuart AC Fiser A Sanchez R Melo F Sali A Comparative protein structure modeling of genes and genomes Annu Rev Biophys Biomol Struct 2000 29 291 325 10.1146/annurev.biophys.29.1.291 10940251

[B104] Sali A Blundell TL Comparative protein modelling by satisfaction of spatial restraints J Mol Biol 1993 234 3 779 815 10.1006/jmbi.1993.1626 8254673

[B105] Petrey D Xiang Z Tang CL Xie L Gimpelev M Mitros T Soto CS Goldsmith-Fischman S Kernytsky A Schlessinger A Using multiple structure alignments, fast model building, and energetic analysis in fold recognition and homology modeling Proteins 2003 53 Suppl 6 430 435 10.1002/prot.10550 14579332

[B106] Meller J Elber R Linear programming optimization and a double statistical filter for protein threading protocols Proteins 2001 45 3 241 261 10.1002/prot.1145 11599028

[B107] Teodorescu O Galor T Pillardy J Elber R Enriching the sequence substitution matrix by structural information Proteins 2004 54 1 41 48 10.1002/prot.10474 14705022

[B108] Tobi D Elber R Distance-dependent, pair potential for protein folding: results from linear optimization Proteins 2000 41 1 40 46 10.1002/1097-0134(20001001)41:1<40::AID-PROT70>3.0.CO;2-U 10944392

[B109] Tosatto SCE The Victor/FRST Function for Model Quality Estimation Journal of Computational Biology 2005 12 10 1316 1327 10.1089/cmb.2005.12.1316 16379537

[B110] Lund O Nielsen M Lundegaard C Worning P CPHmodels 2.0: X3 M a Computer Program to Extract 3 D Models Abstract at the CASP5 conference A102 2002

[B111] Bennett-Lovsey RM Herbert AD Sternberg MJ Kelley LA Exploring the extremes of sequence/structure space with ensemble fold recognition in the program Phyre Proteins 2008 70 3 611 625 10.1002/prot.21688 17876813

[B112] Nicholas K Nicholas H Deerfield D GeneDoc: Analysis and Visualization of Genetic Variation EMBNEWNEWS 1997 14

[B113] Bowie JU Luthy R Eisenberg D A method to identify protein sequences that fold into a known three-dimensional structure Science 1991 253 5016 164 170 10.1126/science.1853201 1853201

[B114] Luethy R Bowie JU Eisenberg D Assessment of protein models with three-dimensional profiles Nature 1992 356 6364 83 85 10.1038/356083a0 1538787

[B115] Sippl MJ Boltzmann's principle, knowledge-based mean fields and protein folding. An approach to the computational determination of protein structures J Comput Aided Mol Des 1993 7 4 473 501 10.1007/BF02337562 8229096

[B116] Xiang Z Soto CS Honig B Evaluating conformational free energies: the colony energy and its application to the problem of loop prediction Proc Natl Acad Sci USA 2002 99 11 7432 7437 10.1073/pnas.102179699 12032300 PMC124248

[B117] Canutescu AA Shelenkov AA Dunbrack RL Jr A graph-theory algorithm for rapid protein side-chain prediction Protein Sci 2003 12 9 2001 2014 10.1110/ps.03154503 12930999 PMC2323997

[B118] Jacobson MP Friesner RA Xiang Z Honig B On the role of the crystal environment in determining protein side-chain conformations J Mol Biol 2002 320 3 597 608 10.1016/S0022-2836(02)00470-9 12096912

[B119] Xiang Z Honig B Extending the accuracy limits of prediction for side-chain conformations J Mol Biol 2001 311 2 421 430 10.1006/jmbi.2001.4865 11478870

[B120] Xiang Z Steinbach PJ Jacobson MP Friesner RA Honig B Prediction of side-chain conformations on protein surfaces Proteins 2007 66 4 814 823 10.1002/prot.21099 17206724 PMC2743384

[B121] Nicholls A Sharp KA Honig B Protein folding and association: insights from the interfacial and thermodynamic properties of hydrocarbons Proteins 1991 11 4 281 296 10.1002/prot.340110407 1758883

[B122] Bashford D Karplus M pKa's of ionizable groups in proteins: atomic detail from a continuum electrostatic model Biochemistry 1990 29 44 10219 10225 10.1021/bi00496a010 2271649

[B123] Gordon JC Myers JB Folta T Shoja V Heath LS Onufriev A H++: a server for estimating pKas and adding missing hydrogens to macromolecules Nucleic Acids Res 2005 33 Web Server W368 371 10.1093/nar/gki464 15980491 PMC1160225

[B124] Myers J Grothaus G Narayanan S Onufriev A A simple clustering algorithm can be accurate enough for use in calculations of pKs in macromolecules Proteins 2006 63 4 928 938 10.1002/prot.20922 16493626

[B125] Pettersen EF Goddard TD Huang CC Couch GS Greenblatt DM Meng EC Ferrin TE UCSF Chimera--a visualization system for exploratory research and analysis J Comput Chem 2004 25 13 1605 1612 10.1002/jcc.20084 15264254

[B126] Kuntz ID Blaney JM Oatley SJ Langridge R Ferrin TE A geometric approach to macromolecule-ligand interactions J Mol Biol 1982 161 2 269 288 10.1016/0022-2836(82)90153-X 7154081

[B127] Richards FM Areas, volumes, packing and protein structure Annu Rev Biophys Bioeng 1977 6 151 176 10.1146/annurev.bb.06.060177.001055 326146

[B128] Ferrin TE Huang CC Jarvis LE Langridge R The MIDAS display system J Mol Graph 1988 6 1 13 27 10.1016/0263-7855(88)80054-7

[B129] Shoichet BK Bodian DL Kuntz ID Molecular docking using shape descriptors J Comp Chem 1992 13 3 380 397 10.1002/jcc.540130311

[B130] Meng EC Shoichet BK Kuntz ID Automated docking with grid-based energy evaluation J Comp Chem 1992 13 505 524 10.1002/jcc.540130412

[B131] Sobolev V Sorokine A Prilusky J Abola EE Edelman M Automated analysis of interatomic contacts in proteins Bioinformatics 1999 15 4 327 332 10.1093/bioinformatics/15.4.327 10320401

[B132] Wallace AC Laskowski RA Thornton JM LIGPLOT: A program to generate schematic diagrams of protein-ligand interactions Prot Eng 1995 8 127 134 10.1093/protein/8.2.127 7630882

